# Emerging Evidence for Intrathecal Management of Neuropathic Pain Following Spinal Cord Injury

**DOI:** 10.3389/fpain.2022.933422

**Published:** 2022-07-28

**Authors:** Jay Karri, James Doan, Christian Vangeison, Marissa Catalanotto, Ameet S. Nagpal, Sheng Li

**Affiliations:** ^1^Division of Pain Medicine, Department of Anesthesiology and Critical Care Medicine, Johns Hopkins University School of Medicine, Baltimore, MD, United States; ^2^Department of Physical Medicine and Rehabilitation, Harvard Medical School, Boston, MA, United States; ^3^Veterans Affairs Boston Healthcare System—West Roxbury Division, Spinal Cord Injury Service, Boston, MA, United States; ^4^Department of Physical Medicine and Rehabilitation, Baylor College of Medicine, Houston, TX, United States; ^5^Department of Orthopaedics and Physical Medicine, Medical University of South Carolina, Charleston, SC, United States; ^6^Department of Physical Medicine and Rehabilitation, McGovern Medical School, University of Texas HSC at Houston, Houston, TX, United States

**Keywords:** spinal cord injury, neuropathic pain, intrathecal drug delivery, glial dysfunction, chronic inflammation

## Abstract

A high prevalence of patients with spinal cord injury (SCI) suffer from chronic neuropathic pain. Unfortunately, the precise pathophysiological mechanisms underlying this phenomenon have yet to be clearly elucidated and targeted treatments are largely lacking. As an unfortunate consequence, neuropathic pain in the population with SCI is refractory to standard of care treatments and represents a significant contributor to morbidity and suffering. In recent years, advances from SCI-specific animal studies and translational models have furthered our understanding of the neuronal excitability, glial dysregulation, and chronic inflammation processes that facilitate neuropathic pain. These developments have served advantageously to facilitate exploration into the use of neuromodulation as a treatment modality. The use of intrathecal drug delivery (IDD), with novel pharmacotherapies, to treat chronic neuropathic pain has gained particular attention in both pre-clinical and clinical contexts. In this evidence-based narrative review, we provide a comprehensive exploration into the emerging evidence for the pathogenesis of neuropathic pain following SCI, the evidence basis for IDD as a therapeutic strategy, and novel pharmacologics across impactful animal and clinical studies.

## Background

Spinal cord injury (SCI) is associated with a plethora of neurological complications including spasticity, sensory deficits, weakness, and neuropathic pain (1–3). While there exist various treatment strategies to treat neuropathic pain in the context of peripheral neuropathy, the treatment of neuropathic pain following SCI is particularly challenging and is a significant contributor to morbidity and suffering (2, 4, 5). In part, this clinical challenge is secondary to an incomplete understanding of the pathophysiological mechanisms underlying this phenomenon and the associated lack of targeted treatment strategies.

While traditionally it was suggested that neuropathic pain is a sequela of injury to somatosensory pathways following SCI, advances from SCI-specific animal studies and translational models in recent years have further enhanced our mechanistic understanding (1, 4, 6, 7). Notably, concepts of neuronal excitability, glial dysregulation, and chronic inflammatory processes have been increasingly recognized (8–11). These developments have also served advantageously to facilitate exploration into the use of neuromodulation as a treatment modality. The use of intrathecal drug delivery (IDD) is a particularly promising neuromodulation intervention as it allows for the introduction of analgesic agents directly into the cerebrospinal fluid (CSF) (12–14). This direct delivery allows for analgesic agents to be administered in a targeted fashion proximal to the dorsal cord, which contains a majority of key pharmacologic targets.

While there has been an extensive emergence of both pre-clinical and clinical research exploring post-SCI neuropathic pain mechanisms and treatments, the comprehensive appraisal and contextualization of this research are largely lacking. In this evidence-based narrative review, we provide a comprehensive exploration into the emerging evidence for the pathogenesis of neuropathic pain following SCI, the rationale for IDD as a particularly beneficial therapeutic strategy, and novel pharmacologic across impactful animal and clinical studies.

## Methods

This review was an evidence-based narrative aimed at characterizing the available evidence exploring intrathecal agents for the treatment of neuropathic pain following spinal cord injury. Both animal model and human studies were included so that we could provide a robust discussion on the mechanisms of neuropathic pain pathogenesis as they translate to the clinical setting. Data sources surveyed include PubMed, Medline, prior systematic reviews, and reference lists surveyed from 1950 through April 2022. All the animal and clinical studies with clearly designated SCI mechanisms, intrathecal interventions, and measured outcomes for analgesia were considered for inclusion. Those studies that included discussion on mechanisms of neuropathic pain after SCI and/or IDD as an intervention were included.

## Results

Utilizing the aforementioned search strategy and study selection process, we identified a total of 25 studies to be included in our evidence-based narrative. These studies were divided into animal model studies (*N* = 15) (Table 1) (15–29) or clinical studies (*N* = 10) (Table 2) (30–39). All the animal studies utilized mice or rat models, of which a majority underwent lower thoracic cord constriction or compression-based SCI. Clinical studies were largely of lower-level data, including one case report along with several case-series or small cohort studies. However, we did identify two randomized controlled studies, although these studies also included rather small cohorts of 11 and 15 patients (Table 2).

**Table 1:** Impactful animal studies exploring novel intrathecal therapies for the treatment of neuropathic pain following spinal cord injury.

**References**	**Study population**	**Intervention**	**Outcomes**
Hosseini et al. (15)	Rats 7 groups *n* = 8 per group SCI Injury: T6-T8 compression	-Injection given 3 days post-induced SCI -Daily intrathecal injection of Muscimol (0.01 μg/10 μl) and/or endomorphin-1 (2/5 μg/10 μl) × 7 days.	-Combination therapy with muscimol and endomorphin-1 significantly increases the pain threshold compared to injection of endomorphin-1 or muscimol alone. -Histological studies showed increased expression of α-2 subunits of GABA receptors, NR1 subunits of NMDA receptors, glutathione, and superoxide dismutase. Concurrently showed decreased malondialdehyde levels on the spinal cord.
Xian et al. (16)	Rats 2 groups: SCI & Sham groups *n* = 15 per group SCI Injury: T10 contusion	-Injection 3 days prior to induced SCI with LV-shNEAT1L, LV-miR-128–3 recombinant lentivirus and its corresponding scrambled control LV-NC	-NEAT-1 affects AQP4 signaling pathway to alleviate the SCI-induced neuropathic pain *via* promoting miR-128–3p expression.
Yao et al. (17)	Rats *n* = 130 SCI injury: T10 constriction	-Intrathecal catheter placed at L5/L6 -Following 2 days recovery period after induced SCI, 4 injections of miR-130a-3p inhibitor and LV-NC & LV-shIGF given at days 0, 4, 8, 12	-Inhibition of miR-130a-3p expression up-regulates the IGF-1/IGF-1R signaling pathway, reducing neuropathic pain in SCI rats.
Shiue et al. (18)	Rats 4 groups *n* = 6 for 2 groups *n* = 3 for 2 groups SCI Injury: L5/L6 spinal nerve ligation	-Intrathecal infusion pump implanted at L5/L6 -At day 3 & 8 post-induced SCI, umbilical cord mesenchymal stem cells (UCMSC) exosome (0.12, 0.6, and 1.2 mg/ml) injected through infusion pump	-A single intrathecal injection of isolated human UCMSC exosomes reversed nerve ligation-induced mechanical and thermal hypersensitivities of the right hind paw of rats at initial and well-developed pain stages. -Continuous intrathecal infusion of exosomes achieved excellent preventive and reversal effects for nerve ligation-induced pain. -Exosomes were associated with inhibition TNF-α and IL-1β activity. Simultaneously increased IL-10, brain-derived neurotrophic factor, and glial cell line derived neurotrophic factor.
Sanchez-Brualla et al. (19)	Rats 2 groups 1. Common peroneal & tibial nerves ligated & transected (SNI, *n* = 16) 2. SCI group (*n* = 32) SCI Injury: Left hemi-section at T8	-Intrathecal catheter placed at L3 -3 treatments: 1. Single injection TCB-2 or water on post-operative day 14 & 21 2. Single injection TCB-1 (0.3 mg/kg) with intrathecal injection of DIOA or vehicle 3. TCB-2 or distilled water intrathecally daily for 7 days, 2 h after injury.	-Up-regulation of KCC2 function by targeting 5-HT2A receptors. This has therapeutic potential in the treatment of neuropathic pain induced by SCI.
Hwang et al. (20)	Rats 2 groups Control and SCI *n* = 24 total SCI Injury: T12 contusive injury	-Intrathecal catheter placed at T10-T11 spinal segment -One time injection of 1,00,000 mESC-NPCs 3 weeks post-SCI -Control group: intrathecal saline injection 3 weeks post-SCI	-The mESC-NPC-derived spinal GABAergic neurons dramatically attenuated the chronic neuropathic pain following SCI. This suggests that the spinal GABAergic mESC-NPCs cultured with low doses of SHH and RA could be alternative cell sources for treatment of SCI neuropathic pain by stem cell-based therapies.
Wang et al. (21)	Rats *n* = 5–6 in each group (14 groups) SCI Injury: spinal nerve ligation of L5 and L6 spinal nerves	-2 groups: intrathecal liposome-encapsulated clodronate (LEC), 2 weeks after injury rats were sacrificed 1 or 5 days later -2 groups: 2 weeks after NS or LEC received intrathecal exenatide for 8 days, analyzed 1 h later -2 groups: intrathecal NS 10 & LEC and 1 day later multiple daily injections of BAA 5 days later -2 groups: intrathecal NS 10 or LEC on day 2—two single intrathecal of morphine and gelsemine 6 h apart -2 groups: intrathecal NS or LEC 20 h before spinal nerve ligation -2 groups: 2 weeks after spinal nerve ligation-−1 intrathecal NS and LEC injection measured 1, 2, 4, 8 h post-injection and 1, 2, 3, 4, 5 days post-injection with MITT assay for microglial viability	-Intrathecal LEC injection significantly attenuated initial (1 day after nerve injury) but not existing (2 weeks after nerve injury) mechanical allodynia. LEC, given intrathecally, is a specific spinal microglial inhibitor and significantly reduces initiation but not maintenance of neuropathic pain, highlighting an opposite role of spinal microglia in different stages of neuropathic pain.
Hama et al. (22)	Rats 4 experiments 1. Muscimol vs. baclofen (*n* = 7 per group) 2. MK-801 vs. vehicle (*n* = 3–7 per group) 3. Baclofen vs. baclofen + ketamine vs. baclofen + SHG (*n* = 5–7 per group) 4. Vehicle vs. vehicle+baclofen vs. CGP 35348+ Vehicle vs. CGP 35348+ Baclofen (*n* = 6 group) SCI Injury: Compression injury at T6-T7	-3 weeks after SCI surgery, intrathecal catheter placed caudal to SCI at level of lumbar enlargement -GABA-B antagonist pre-treatment (4 groups) -Analyzed 30 min post-intrathecal injection & 120 min post-intrathecal injection and peak anti-nociceptive of each med/combo was found	-Blocking spinal NMDA receptors alone is not sufficient to ameliorate SCI hypersensitivity. -Simultaneous activation of spinal GABA-B receptors and NMDA receptor blockade with ketamine, leads to significant antinociception. -Adverse effect: Psychomotor SE with MK-801
David et al. (23)	Rats 3 groups 1. IL-1β (*n* = 12) 2. TNF-α (*n* = 7) 3. CXCL1, CpG ODN 1,826 (*n* = 8) SCI Injury: T8 severe contusion Injury	-Intrathecal administration at L5-L6 24 h after injury and repeated every 48 h	-Intrathecal administration of a TLR9 antagonist, cytidine-phosphate-guanosine oligodeoxynucleotide 2088 (CpG ODN 2088) to mice sustaining a severe contusion SCI, diminishes injury-induced heat hypersensitivity. -Proved there was a weakened inflammatory reaction by finding a decrease in the number of CD11b-, CD45- and CD3-immunoreactive cells and a reduction in tumor necrosis factor-α (TNF-α) expression at the epicenter.
Hajimashhadi et al. (24)	Rats 3 groups 1. Intact (no surgical intervention) 2. Sham (laminectomy no SCI) 3. SCI ± vehicle vs. [Pyr^1^] apelin-13 *n* = 8 rats per group SCI Injury: Compression at T7-T8	Intrathecal catheter placed at T7-T8 Experimental SCI group were administered volume of 10 μl Vehicle (NS) 1 μg [Pyr^1^] apelin-13 5 μg [Pyr^1^] apelin-13 All interventions once a day for × 1 week from day 1 post-SCI	-Found that rats treated with both 1 μg and 5 μg [Pyr^1^] apelin-13 had improvement in mechanical allodynia and thermal hyperalgesia in a dose dependent manner. -Microscopic analysis of spinal cord in rats treated with [Pyr^1^] apelin-13 showed less necrosis in the area of SCI compared to controls. -Spinal cord in rats treated with [Pyr^1^] apelin-13 had less caspase-3 (pro-apoptotic enzyme contributing to spinal cord damage) expression.
Yu et al. (25)	Rats 3 groups 1. SCI group + vehicle 2. SCI group + bolus adm [-] huperzine A (HUP-A) 3. SCI group + continuous adm HUP-A *n* = 3–4 per group SCI injury: compression T10	Spinal cord osmotic pump implanted into the intrathecal space at L5-L6 HUP-A dosage was determined based on whether subject received bolus vs. continuous infusion	-SCI rats treated with both bolus and continuous HUP-A demonstrated decreased hyperalgesia determined by paw withdrawal times to force. -Neuropathic pain reduction believed to be due to the cholinergic effects of HUP-A inhibiting activation of macrophages, microglia and astrocytes in CNS.
Avila-Martin et al. (26)	Rats 6 groups 1. Uninjured control (*n* = 8) 2. 0.9% saline treatment (*n* = 8) 3. Albumin treatment (*n* = 8) 4. Oleic Acid treatment (*n* = 8) 5. Albumin-Oleic Acid treatment (*n* = 11) 6. Albumin-Elaidic Acid treatment (*n* = 11) SCI injury: T9 contusion injury	Intrathecal catheter inserted below T9 contusion site Delivered assigned treatment immediately following SCI and every 3 days after for total 28 days	-Rats treated with albumin-oleic acid mixture had greatest recovery of gross motor function and greatest inhibition of tibialis anterior reflex activity (measure of neuropathic pain). -Histochemical analysis showed an increase in serotonin density below the level of the injury in rats treated with albumin-oleic acid mixture. Serotonin is a known mediator in neuropathic pain.
Lv et al. (27)	Rats 6 groups 1. Control group 2. SCI no treatment 3. Early-rapamycin group 4. Early-vehicle group with intrathecal injection of DMSO 5. Late-rapamycin group 6. Late-vehicle group with intrathecal injection of DMSO *n* = 5 per group SCI injury: Constriction injury	The “early” intervention groups was given treatment 4 h after SCI The “late” intervention group was given treatment 7 days after SCI -For each rat given treatment, they received Rapamycin OR DMSO injections daily for 3 consecutive days	-Groups treated with early and late rapamycin had a statistically significant increase in mechanical and thermal tolerance compared to DMSO treated rats. -Intrathecal injection of rapamycin weakens constricted cord injury associated hyperalgesia by inhibiting activation of astrocytes.
Roh et al. (28)	Rats 4 groups: 1. Vehicle treated 2. 1 μg Carbenoxolone (CARB) 3. 5 μg CARB 4. 25 μg CARB *n* = 5 per group SCI injury: Hemi-section at T13	Direct transcutaneous intrathecal injection Each rat was given 10 μl of its assigned treatment twice per day from post-op days 0–5 (induction phase) and post-operative days 15–20 (maintenance phase)	-Administration of CARB only during the induction phase (days 0–5) had improvement of below level neuropathic pain in a dose dependent manner. -Administration of CARB 15 days after injury did not improve neuropathic pain.
Martini et al. (29)	Mice and Rats 2 groups: 1. Vehicle 2. 300 pmol of lipoxin A4 (LXA4) SCI injury: Left hemi-section at T10	Intrathecal catheter placed at L4-L5 level Each subject received 10 μl of assigned treatment at 4 and 24 h post-SCI	-There was significant improvement of mechanical allodynia by day 7 in subjects treated with LXA4 in the contralateral paw. Mechanical allodynia found to improve in the ipsilateral paw by day 14. Effects lasted for 35 days. -Histochemistry examination of the thoracic spine taken from subjects at day 36 showed a decrease in IBA-1 density which is associated with microglial activation, contributing to neuroinflammation.

**Table 2:** Impactful clinical studies exploring novel intrathecal therapies for the treatment of neuropathic pain following spinal cord injury.

**References**	**Study population**	**Intervention**	**Outcomes**	**Adverse effects**
Brinzeu et al. (30)	Human -SCI traumatic (*n* = 16), ischemic (*n* = 2), syringomyelia (*n* = 2) -11 out of 20 patients had implanted permanent pumps -Injuries ranged from C2-L2	-3 lumbar punctures at 72 h intervals -Administer ziconotide boluses at increased doses (0.5, 1.0, 1.5 μg diluted in 2 ml of saline) -Catheter implanted 2–3 levels above lesion level with doses increased 0.5 μg per visit (at 1, 3, 6, 8, 12 month follow up) with a max of 20 μg/day	-55% responded to the test and 40% benefited from long term treatment with a clinically significant impact on pain. Average follow up 3.59 years ± 1.94 years. -IT ziconotide is a possible alternative for the treatment of pain in patients with SCI & below level neuropathic pain.	-3 patients with severe AE (2 increase in CPK & 1 acute urinary retention)
Kumru et al. (31)	Human -Randomized control trial of SCI patients -*n* = 11	-Intrathecal baclofen bolus 50 μg at L3/L4 level, increased dose 100 μg 1 week later if prior dose didn't relieve pain -Placebo: 1 ml of NaCl at same location	-Clear analgesic effects of a single ITB bolus on all subtypes of neuropathic pain (continuous and paroxysmal pain, allodynia, dysesthesias). -Patients experienced significantly less interference of neuropathic pain with activities of daily living over 24 h period post-injection.	None
Vaquero et al. (32)	Human -SCI at cervical (4) thoracic (4) lumbar (3) -AIS A (3) B (4) C (4) *n* = 11	-3 intrathecal autologous MSC's (100 × 10^6^) injections from blood and bone marrow aspirate -Lumbar puncture at initial visit, month 4, month 7 and follow up at 10 months	-10 month follow up after autologous MSCs proved variable clinical improvements in neuropathic pain regardless of the level of injury, degree of injury, age or time elapsed from SCI. -3 patients, classified ASIA A, B, and C changed to ASIA B, C and D, respectively.	-Sciatic pain (37.5%), headaches and pain in area of LP, one severe AE unrelated to tx that necessitated withdrawal from study
Kumru et al. (33)	Humans -SCI case series -Patient 1 C6 AIS A (*n* = 1) -Patient 2 T11 AIS C (*n* = 1) -Patient 3 C4 AIS D (*n* = 1)	-Intrathecal baclofen pump placed -Study does not specify level of implant or dose administered	-Patient 1: at 6 months, neuropathic pain improved 70% with ITB dose of 265 μg. -Patient 2: at 7 months, 60% decrease in neuropathic pain. -Patient 3: at 7 months, 80% improvement in neuropathic pain with ITB dose of 600 μg.	None
Vaquero et al. (34)	Humans -Chronic SCI with average of 3–44 years post-injury (*n* = 10) SCI injury: cervical (5) thoracic (2) lumbar (3)	-100 million MSCs into subarachnoid space by lumbar puncture (month 1 of the study) -Repeated at months 4 and 7 until reaching a total dose of 300 million MSCs, follow up at 10 months	-Significant and progressive improvement in neuropathic pain intensity after the first administration of MSCs. -Study suggests benefit of intrathecal administration of autologous MSCs for the treatment of neuropathic pain in patients with SCI.	None
Siddall et al. (35)	Human Double blinded RCT *n* = 15 *Main qualifications:* patients with neuropathic pain following SCI failing all other pharmacotherapy management, injury had to be sustained >4 weeks ago, injury had to be below C4	-Intrathecal catheter within lumbar region -Patients received injection of either saline, morphine, or clonidine -Part I: Patient received daily dose x3 days -Part II: Each patient received mixture of morphine and clonidine -7 subjects underwent blood and CSF sampling at L3-L4 and C7-T1 level to evaluate drug migration	-Combination of morphine and clonidine produced statistically significant pain relief (63% pain relief from baseline) 4 h after administration. Morphine or clonidine alone did not produce significant pain relief. -Study is suggestive that morphine and clonidine delivered together intrathecally have synergistic effects. -Intrathecal administration of agents should be above the level of injury, especially if obstruction of CSF flow is a question, to allow for better distribution of agent into CSF. -Study suggests that this intervention is best for treating at-level neuropathic pain vs. below-level pain.	**Morphine intrathecal injection:** pruritus (38%), O_2_ desaturation (50%), sedation (50%), nausea (13%), hypotension (6%) **Clonidine intrathecal injection:** hypotension (53%), nausea (40%), sedation (33%), O_2_ desaturation (33%) and dry mouth (20%) **Morphine and clonidine mixture intrathecal injection:** hypotension (56%), O_2_ desaturation (44%), pruritus (25%), dry mouth (25%) and sedation (3%)
Kumru et al. (36)	Human -SCI patients with severe spasticity (SCI level ranged C4-C10) -Control group = 9, age and gender matched healthy adults *n* = 11	-1 time 50 μg injection of intrathecal baclofen at L3/L4 level	-Self-reported decrease of neuropathic pain by subjects was NOT significant 4 h after injection. - Following intrathecal baclofen patients had increase in heat pain perception threshold at 1, 2 and 4 h. -Following intrathecal baclofen patients had a decreased in evoked heat pain perception at 2 and 4 h.	None
Saulino et al. (37)	Human Case report -23 year old female T5 ASIA A -Injury date = 14 years prior to study	-Intrathecal catheter placed at T7 -Continuous intrathecal infusion of hydromorphone: final dose of 8.6 mg/day -Continuous intrathecal infusion of ziconotide: final dose of 10 mcg/day -Continuous Hydromorphone and Ziconotide mixture: Most effective dose of 1.32 mg hydromorphone daily and 11 mcg of ziconotide	-Combination of intrathecal hydromorphone and ziconotide improved both at level and below level neuropathic pain for at least 15 months. -Hydromorphone alone only improved at level pain. -Ziconotide alone only improved below level pain. -Following initiation of hydromorphone and ziconotide intrathecal infusion patient's daily oral opiate use decreased.	-Hydromorphone infusion led to transient nausea and constipation
McCormick et al. (38)	Human -SCI with spasticity *n* = 38	-Mean dose of oral baclofen: 86 mg/day -Mean dose of intrathecal baclofen: 577 μg/day -Study used self-assessment questionnaires	-Study showed no statistical significance for reduction in pain between oral and intrathecal baclofen use.	None
Saulino et al. (39)	Human 2 Groups -Intrathecal baclofen (ITB) and intrathecal morphine groups (*n* = 47) -ITB monotherapy (*n* = 136) 75% of the patients involved in this study had SCI	-Average dose of ITM = 1,730 μg/day -Severity of pain assessed using visual analog scale of pain intensity	-Addition of intrathecal morphine to intrathecal baclofen infusion decreased pain reported by subjects. Pain was assessed using visual analog scale of pain intensity. -30 out of 47 patients had a greater than 30% decrease in reported pain. -13 out of 47 patients had a greater than 50% decrease in pain.	-8 out of 47 patients experienced adverse events associated with ITM including cognitive dysfunction, sedation, constipation

## Discussion

### Pathogenesis of Neuropathic Pain Following SCI: Established Evidence and Novel Understanding

Approximately 50% of individuals with SCI develop neuropathic pain, which is largely subdivided into above-level, at-level, or below-level pain presentations (4, 5, 7, 40). At-level neuropathic pain is the most prevalent subset and is designated by pain within one dermatome rostral and three dermatomes caudal to the neurological level of injury. A small subset of patients has below-level pain, which often affects the lower extremities in a more gradual pattern with less dysesthesia and allodynia than reported for at-level pain.

When attempting to understand the physiologic and mechanistic pathways leading to the emergence of neuropathic pain after a spinal cord injury, many established theories are rooted from studies of animal models of the peripheral nerve injury that originated in the 1950s (1, 4, 7, 40–43). This research, however, is limited given that the spinal cord contains a complex network of several tissue types, the vast majority of which are non-neural (44–46). Histological sections of the human spinal cord have found that the dorsal segments contain ~10-fold the density of glial cells relative to neurons (44). More nuanced animal studies utilizing animal models of SCI have been utilized in recent years and concordantly, there has been an emergence in the recognition of neuroimmune and glial dysregulation in facilitating neuropathic pain.

Studies from the early 1990s first demonstrated the presence of increased astrocyte density in the spinal dorsal horns as visualized by GFAP immunostaining (47, 48). More interestingly, this also correlated with hyperalgesia symptoms after a sciatic nerve compression. Subsequently, it was also demonstrated that glial activation led to hyperalgesia and inflammation in correlation with TNF-α. In the early 2000s, rat studies showed that rats with a thoracic 13 (T13) unilateral hemisection of the spinal cord produced microglia activation with TNF-α expression noted below the level of the lesion, which evidently corresponded with allodynia of the rat's hindpaw (9, 49, 50). Furthermore, when the rat with the T13 hemisection of the spinal cord was treated with a TNF-α blocker, such as etanercept, there was decreased mechanical allodynia displayed and decreased microglial activation. When specifically targeted with a microglial inhibitor, such as minocycline, the rat's pain behaviors were also improved, which showed that TNF-α in association with microglia played a critical role in the emergence of neuropathic pain after a spinal cord injury. Even more recently, inducible dysregulation macrophages have been shown to amplify chronic inflammation to facilitate pain and prevent neuronal regeneration (51–53). This diminished neuronal regeneration has been thought to counteract the productive benefits of spinal plasticity, which may advantageously dampen intraspinal pain circuitry (53–55). Just as importantly, it has been shown that this inducible inflammatory dysregulation and pain modulation may selectively occur at the spinal level, and not within supraspinal centers (55).

By taking a closer look into the interactions of these immune modulators, we can gain further understanding on the development of neuropathic pain after a spinal cord injury. Following SCI, nociceptors sensitize glial modulators, which include ATP, colony-stimulating factor-1 (CSF-1), chemokines, and cascade-6 (CASP6) (9, 48, 49). These modulators then activate the spinal microglia in the dorsal horns, thereby leading to increased expression of CX3CL1 receptors for the Fractalkine ligand for recruitment of immune cells and development of neuroinflammation. Microglia also enhance secretion of TNF-α and IL-1β, causing increased excitatory synaptic transmissions with decreased inhibitory transmissions of somatosensory information (including nociceptive) of the lamina II region of the spinal cord. Recent studies have also shown that activation of astrocytes in the dorsal horns causes an increased release of nerve growth factor (NGF), which leads to neuropathic pain (8, 9, 45–48). Thus, SCI causes activation of astrocytes and microglia that may extend longitudinally across the dorsal cord and facilitate neuropathic pain transmission in above-level, at-level, and below-level phenotypes.

When an SCI occurs, astrocytes usually respond first during the immune response and become reactive, leading to an increased release of cytokines (such as IL-1β, IL-6, TNF-α, and interferon-γ) and chemokines, which in turn leads to recruitment of neutrophils and pro-inflammatory macrophages (9, 48, 49). The adaptive immune response (including T- and B-lymphocytes) also contribute to the inflammatory response after a SCI which usually peaks in number around 1 week after injury and remains elevated during the chronic period. These processes of the immune response contribute to the notion of spinal neuroinflammation as a mechanism for the development of neuropathic pain after SCI.

### Targeted Pharmacological Management: the Rationale for Intrathecal Drug Delivery

In addition to the neuropathic pain that develops as a consequence of glial activation and spinal neuroinflammation, there is also a neuronal dysregulation component that contributes to the development of neuropathic pain (8–11). Post-traumatic neuronal hyperexcitability and increased spontaneous activity in the dorsal root ganglion and spinal nerves correlate to noxious dysesthetic responses to chemical, mechanical, and thermal stimulation (1, 4, 5, 8–11). Nociceptive sensory neurons including myelinated A-delta fibers and unmyelinated C-fibers transmit ascending sensory input to supraspinal areas, including the brainstem and thalamus (8–11, 40). The transmitted nociceptive input is processed, and the midline relay is located at the periaqueductal gray and rostral ventromedial medulla before traveling down the descending pathways associated with the spinal cord. The usual modulators released as a communication process of the primary nociceptors include glutamate, ATP, neuropeptides (such as calcitonin gene-related peptide and substance P), chemokines (such as CCL2, CCL21, and CX3CL1), cytokines (such as IL-1β, IL-6, and TNF-α), and growth factors (such as BDNF, neuregulin 1, and basic fibroblast growth factor).

Irrespective of the precise mechanisms of microglial activation or spinal neuroinflammation, a majority of post-SCI noxious pathogenesis is thought to derive as a consequence of injury to somatosensory pathways. The delivery of spinal analgesics *via* IDD attenuates the transmission of pain pathways directly at the level of the dorsal columns, where noxious sensitization occurs (12–14). Consequently, IDD is a highly favorable pharmacologic delivery system given that most enteric medications fail to reach sufficient cerebrospinal fluid concentration and confer significant systemic side effects at higher dosages. Currently, the only FDA-approved medications for intrathecal analgesia include morphine and ziconotide. While not approved, other medications including bupivacaine, clonidine, and other opioids are supported for clinical use by the polyanalgesic consensus conference guidelines (12–14, 56). While all of these medications act largely to attenuate ascending somatosensory transmission and/or activate descending inhibitory pathways, medications to treat microglial activation and spinal neuroinflammation are lacking. As the importance and roles of these more novel mechanisms are increasingly recognized, we expect an emergence of targeted pharmacotherapies, including those that may be delivered intrathecally.

The pharmacokinetics of intrathecal agents are not fully understood but are largely thought to involve various parameters specific to the medication and catheter (12, 14). The major medication parameters are the drug baricity, the density relative to CSF, and octanol-water partition coefficient, the measure of lipophilicity (12–14, 56). Lipophilic agents, such as bupivacaine and fentanyl, have limited spread within the CSF and thereby may prove particularly advantageous for treating at-level neuropathic pain conditions. On the contrary, more diffuse pain syndromes may be better served with the use of hydrophilic agents. Similarly, hyperbaric agents demonstrate greater CSF spread relative to isobaric and hypobaric agents. With regard to catheter parameters, the tip location heavily influences medication response. Given that epidemiologically most patients with SCI have cervical level injuries, IDD catheters at the cervical level may prove most beneficial when attempting to use hyperbaric and lipophilic agents to treat at-level neuropathic pain (1–3, 12–14). However, the adverse effect associated with cervical catheter placement include medication diffusion to respiratory centers, leading to respiratory depression with opioid medications. Consequently, the catheter tip location must represent a judicious decision in accordance with the mono- or polyanalgesic pharmacotherapies utilized.

### Future Promise of Intrathecal Drug Delivery: Novel and Emerging Pharmacotherapies

In patients with SCI, a reduction in inflammatory processes has been associated with a reduction of neuropathic pain. Common inflammatory markers, such as IL-6, IL-1β, and TNF-α, have all been known to increase following SCI, thereby, contributing to central nervous system inflammation (4, 18). These markers have differing roles in the inflammatory cascade and work by enhancing mechanical and heat hypersensitivity reactions that are measures of neuropathic pain in animal models. Animal studies have shown that reducing these inflammatory markers can be a promising mechanism to decrease neuropathic pain; however, this has not been tested in human subjects (18, 23, 57). For example, exosomes isolated from human umbilical mesenchymal stem cells have the ability of inhibiting TNF-α, IL-1β, while simultaneously increasing IL-10, an innate anti-inflammatory interleukin, when continuously infused into the intrathecal space (18). Pro-inflammatory pathways have been selectively targeted, such as toll-like-receptors (a component of the innate immune response), caspase-3 enzymes (proteolytic enzymes playing a role in apoptosis), and the ALX/FPR2 pathway (which modulates microglial activation) resulting in a decrease in neuropathic pain in rat subjects. As aforementioned, there are different pathways to evoke an anti-inflammatory response which has been proven to play a role in decreasing neuropathic pain in spinal cord injury. It is through these pathways that spinal cord inflammation can be reduced, leading to a decrease in neuropathic pain in rat subjects (23, 24, 29). In the study that targeted the ALX/FPR2 pathway with an endogenous lipid mediator, lipoxin A4, rodents demonstrated decreased mechanical allodynia for 35 days (29).

In addition, stem cell biologics display promise in improving neuropathic pain. Traditional stem cell therapy works to enhance local inflammation in tissues with limited vascularity to aid in healing. However, when used in spinal cord injury it can allow researchers to introduce known anti-neuropathic mediators directly to the site of injury. For example, mouse embryonic stem cell-derived neural pre-cursor cells (mESC-NPC) composed of GABAergic neurons were administered intrathecally 3 weeks after induced SCI; this resulted in a statistically significant reduction of neuropathic pain in rodents measured by mechanical allodynia and vocalization at the end of the 7-week threshold (20). Other studies have shown that persistent neuropathic pain following SCI is due to a loss of GABA inhibitory influences on the spinal dorsal horn neurons. Vaquero et al. introduced this idea to patients with SCI and injected 100 million autologous mesenchymal stromal cells (MSC) at three different times post-injury. At the 10-month follow-up, patients reported significant improvement in neuropathic pain (34). In another study with 11 patients with SCI with different incomplete AIS classifications, Vaquero et al. (32) was again able to further exhibit that patients given intrathecal MSC had a variable response in a reduction in the neuropathic pain. Since the study of MSC is in its infancy, further study is required, particularly with a focus on adverse effects and optimizing dose-benefit profiles.

A smaller subset of experimental treatments has been explored looking at the gene-based therapies aimed at producing nociceptive effects on the neuropathic pain. The wide breadth of the possibilities with these therapies has given researchers the ability to manipulate upstream and downstream genes that work to directly contribute to neuropathic pain. Animal rat studies inhibiting the expression of a specific microRNA allowed for the insulin-like growth factor signaling pathway (IGF-1/IGF-1R) to be upregulated, mitigating neuronal apoptosis and microglial activation (17). On the other hand, promoting the expression of long non-coding RNA variants, such as miR-128–3p, attenuates inflammation and clinically correlates with a reduction of neuropathic pain (16). The versatile utility of gene-based therapies has not been trialed with human subjects but has great future promise. Most importantly, within the studies performed utilizing gene-based therapies, no adverse effects have been documented within the rodent population.

As aforementioned, the only FDA-approved spinal analgesics include ziconotide and morphine. Intrathecal baclofen (ITB) is FDA approved for use in spasticity management, however, and interestingly may prove beneficial as an analgesic compound separate from its spasmolytic properties (31, 33, 36). When delivered intrathecally, baclofen works as a GABA-B analog and can target neurons in the dorsal horns with high-density GABA-B receptors. Neuropathic pain has been believed to be a result of neuronal hyperexcitability in this region due to loss of GABA inhibition. Oral baclofen has no statistically significant evidence for the reduction in neuropathic pain compared to ITB (38). However, these differences in analgesic capacity may be secondary to pharmacokinetic disparities given that conventional enteral baclofen doses reach minimal concentrations in the CSF.

The utility of ITB as an analgesic compound has been evidenced by the high -level randomized control trials. In these trials patients had reductions in neuropathic pain and improvements in their activities of daily living within 24 h post-injection of ITB (31). There does exist a great difficulty in characterizing ITB's analgesic-specific benefits given that the treatment of spasticity is a significant confounder. A recent review by Karri et al. (13) explored this phenomenon and concluded that ITB may be an effective analgesic agent independent of its spasmolytic effects. Although the evidence basis reviewed was largely vague, given the absence of clearly defined pain and spasticity outcome measures. Nonetheless, ITB was deemed to be a relatively safe and particularly beneficial therapy that warrants consideration in those patients with SCI with severe spasticity as well as neuropathic pain. Although, careful clinical surveillance and follow-up are prudent in this population given that ITB withdrawal specifically can prove fatal, unlike with withdrawal phenomena to other intrathecal agents including opioids and ziconotide.

## Conclusion

Advances from animal studies and translational models continue to demonstrate that neuropathic pain following SCI involves complex pathogenesis that includes neuronal excitability, glial dysregulation, and chronic inflammation. While currently utilized intrathecal analgesic agents provide analgesic benefits, targeted treatments to modulate underlying pathogenesis are largely lacking. However, there exists increasingly recognized research supporting the promise of intrathecal immunomodulators, stem-cell-based treatments, and even genetic therapies for use in the chronic pain treatment. In addition, the use of ITB for analgesic indications is not approved but appears to have some rationale in patients with SCI. Given the benefits of targeted, site-specific pain treatment, IDD is a particularly beneficial treatment strategy in appropriate individuals and its benefits will only be commensurate with forthcoming novel pharmacologics.

## Author Contributions

JK, AN, and SL conceived the idea and objectives of the project, made final revisions, and prepared the manuscript for submission. JK, JD, CV, and MC participated in literature review and principle authorship. All authors meet the ICMJE criteria for authorship, take responsibility for the integrity of this work, and have given their approval for publication.

## Conflict of Interest

The authors declare that the research was conducted in the absence of any commercial or financial relationships that could be construed as a potential conflict of interest.

## Publisher's note

All claims expressed in this article are solely those of the authors and do not necessarily represent those of their affiliated organizations, or those of the publisher, the editors and the reviewers. Any product that may be evaluated in this article, or claim that may be made by its manufacturer, is not guaranteed or endorsed by the publisher.
